# Impact of Image Preprocessing and Crack Type Distribution on YOLOv8-Based Road Crack Detection

**DOI:** 10.3390/s25072180

**Published:** 2025-03-29

**Authors:** Luxin Fan, Saihong Tang, Mohd Khairol Anuar b. Mohd Ariffin, Mohd Idris Shah Ismail, Xinming Wang

**Affiliations:** Faculty of Engineering, Universiti Putra Malaysia UPM, Serdang 43400, Selangor, Malaysia

**Keywords:** YOLOv8, crack detection, image preprocessing, deep learning, dataset balancing, pavement inspection, computer vision

## Abstract

Road crack detection is crucial for ensuring pavement safety and optimizing maintenance strategies. This study investigated the impact of image preprocessing methods and dataset balance on the performance of YOLOv8s-based crack detection. Four datasets (CFD, Crack500, CrackTree200, and CrackVariety) were evaluated using three image formats: RGB, grayscale (five conversion methods), and binarized images. The experimental results indicate that RGB images consistently achieved the highest detection accuracy, confirming that preserving color-based contrast and texture information benefits YOLOv8’s feature extraction. Grayscale conversion showed dataset-dependent variations, with different methods performing best on different datasets, while binarization generally degraded detection accuracy, except in the balanced CrackVariety dataset. Furthermore, this study highlights that dataset balance significantly impacts model performance, as imbalanced datasets (CFD, Crack500, CrackTree200) led to biased predictions favoring dominant crack classes. In contrast, CrackVariety’s balanced distribution resulted in more stable and generalized detection. These findings suggest that dataset balance has a greater influence on detection accuracy than preprocessing methods. Future research should focus on data augmentation and resampling strategies to mitigate class imbalance, as well as explore multi-modal fusion approaches for further performance enhancements.

## 1. Introduction

The Pavement Management System (PMS) plays a crucial role in ensuring safety and preventing structural failures. With the advent of Industry 4.0, intelligent and data-driven solutions have rapidly developed across various domains, bringing new opportunities for the PMS. In the PMS, advancements in computer vision and deep learning (DL) have enabled automatic road crack detection based on DL models. By leveraging automated detection, the PMS can promptly acquire road damage information, formulate appropriate maintenance plans, reduce manual intervention, and enhance detection efficiency. Consequently, the maintenance and management of transportation infrastructure are shifting gradually from traditional manual inspections to intelligent automation.

Crack detection algorithms such as You Only Look Once (YOLO) [1], Faster Region-based Convolutional Neural Network (Faster R-CNN) [2], and U-Net [3] have emerged in response to this need. YOLO outperforms two-stage detectors such as Faster R-CNN in terms of inference speed and is more suitable for real-time road inspection applications. Meanwhile, U-Net has demonstrated strong feature learning capabilities and often requires significantly higher computational resources. Thus, it is less feasible for real-time crack detection in field conditions. As an evolution of the YOLO framework, YOLOv8 [4] introduces several improvements, including enhanced detection accuracy, an optimized network structure, and better generalization capabilities. Compared to its predecessors, YOLOv8 integrates improved feature extraction and a more efficient loss function, making it particularly suitable for detecting fine-grained structural defects such as road cracks. Recent advancements, such as YOLOv9 [5], have demonstrated a strong performance in object detection tasks. However, YOLOv8 remains a compelling choice for real-time applications due to its efficiency and relatively lower computational cost [6]. Given these advantages, this study employed YOLOv8s as the primary detection model for road crack analysis.

The performance of YOLO-based models depends largely on the quality of input images and the composition of the dataset. Previous studies have explored various image preprocessing techniques to enhance crack detection performance. For instance, Tong et al. proposed converting RGB images into grayscale and extracting crack attributes using k-means clustering analysis, which improved detection accuracy when combined with quadrature encoding and stochastic gradient descent [7]. Hou et al. adopted a binary black-and-white transformation approach, which simplified feature representation and addressed the imbalance of crack types by expanding the dataset through augmentation, ultimately improving detection accuracy [8]. Similarly, Chun et al. employed a recursive approach where misclassified samples were collected and used for retraining, enhancing model robustness [9].

Beyond preprocessing techniques, dataset composition significantly influences crack detection performance. Du et al. constructed a dataset with road crack images collected under varying weather and lighting conditions, demonstrating that such diversity enhances model adaptability [10]. Maniat et al. explored the feasibility of extracting pavement images from Google Street View (GSV) for dataset creation, showing its effectiveness in real-world scenarios [11]. Additionally, Fan et al. highlighted that shadows can resemble cracks, which may interfere with detection; thus, incorporating shadow variations in training data and applying shadow removal techniques can significantly improve detection accuracy [12]. Despite these advancements, there remains a gap in systematically evaluating the impact of different image preprocessing techniques and dataset balance on the performance of YOLO-based road crack detection models.

This study aimed to systematically investigate the impact of image preprocessing and crack type distribution balance on the performance of YOLO-based road crack detection. To achieve this, the following key objectives were defined:

- Construct a self-built dataset, CrackVariety, and incorporate publicly available datasets, including the CrackForest Dataset (CFD) [13], Crack500 [14], and CrackTree200 [15], for comparative analysis.
- Convert the RGB images from all four datasets into grayscale and binarized formats, conducting comparative experiments to determine the type of image which is most suitable for road crack detection.
- Re-annotate all datasets by refining crack classification into longitudinal cracks (LCs), transverse cracks (TCs), alligator cracks (ACs), oblique cracks (OCs), and potholes (PHs).
- Utilize CrackVariety, which maintains a balanced distribution of crack types, to analyze the effects of balanced vs. imbalanced crack type distributions on model accuracy and generalization.

The main contributions of this study are as follows:

- Comprehensive evaluation of image preprocessing techniques (RGB, grayscale, binarization) in YOLO-based road crack detection, providing insights into the optimal input format.
- Development of a balanced crack dataset (CrackVariety) with detailed crack type classification, ensuring equal representation of various crack types.
- Re-annotation of existing datasets (CFD, Crack500, CrackTree200) with refined crack labels, improving dataset consistency and classification accuracy.
- Empirical analysis of crack type distribution balance, demonstrating its impact on model accuracy and generalization performance.

## 2. Datasets and Methods

### 2.1. Datasets

Over the past decade, significant progress has been made in image-based road defect detection, promoting the development of various publicly available datasets. This section provides an overview of publicly available datasets in pavement crack detection, along with the datasets selected for this study.

CrackTree200, introduced by Zou et al. [15] in 2012, contains 206 road images with a resolution of 800 × 600. It captures cracks under low contrast, uneven lighting, and shadow interference. However, this dataset does not explicitly classify crack types.

The CFD dataset, proposed by Shi et al. [13] in 2016, consists of 118 images representing urban road conditions in Beijing, China. These images are captured using an iPhone 5 with a resolution of 480 × 320 and feature manual crack annotations but do not categorize cracks into specific types. Additionally, they contain noise factors such as shadows, oil stains, and water puddles.

The GAPs384 (German Asphalt Pavement Distress) dataset is a high-quality dataset for pavement distress detection, developed by the Neuroinformatics and Cognitive Robotics Laboratory at Ilmenau University of Technology, Germany [16]. It consists of 1969 grayscale images with a resolution of 1920 × 1080 pixels. The dataset includes various types of pavement distress, among which cracks are categorized into longitudinal, transverse, and ACs.

DeepCrack consists of 537 RGB images with a resolution of 544 × 384. It was designed for evaluating crack detection models in multi-scale and multi-scene environments, ensuring dataset diversity. Cracks in this dataset are categorized based on width rather than structural type [17].

Crack500, introduced by Yang et al. [14] in 2019, includes 500 RGB images with a resolution of 2000 × 1500, which were captured using mobile devices at Temple University’s main campus. Like CFD, this dataset does not provide explicit crack type classification.

The EdmCrack600 dataset contains 600 fully pixel-annotated crack images, collected using a GoPro Hero 7 Black action camera. The dataset covers various real-world pavement conditions, including sunlight variations, shadows, occlusions, and diverse road textures. The image resolution is 1920 × 1080, but no crack type classification is provided [18].

In recent years, CFD, Crack500, and CrackTree200 have been widely used as benchmark datasets for validating deep learning-based crack detection models [19]. These datasets were selected in this study because they cover diverse road environments and they are frequently used in deep learning research, allowing for performance comparison with existing studies. Since these datasets lack detailed crack type classifications, this study re-annotated the crack labels to ensure consistency across datasets. Existing datasets provide valuable benchmarks. One of the notable limitations is the imbalanced crack type distributions.

In order to have balanced crack type distributions, we constructed CrackVariety, which is a publicly available dataset of images collected using a Huawei Mate40 Pro (Huawei Technologies Co., Ltd., Shenzhen, China at University Putra Malaysia (UPM). The dataset comprises 400 high-resolution images (3072 × 4096) for ensuring detailed feature extraction for crack detection. To enhance model generalization and minimize dataset bias, CrackVariety maintains an equal distribution of five crack types (LCs, TCs, ACs, OCs, and PHs), namely 80 images of each type.

In addition to crack type diversity, CrackVariety incorporates varied environmental conditions. The various environmental conditions are sunny and rainy weather, shadows, oil stains, water puddles, and occlusions, which reflect real-world pavement scenarios. The dataset was annotated by using LabelImg1.8.6 with bounding box annotations applied to each crack region.

For the self-built CrackVariety dataset, all images were captured using a Huawei Mate40 Pro from a fixed height of approximately 1.5 m above the pavement surface. Given the camera specifications and shooting conditions, the field of view corresponds to a ground coverage of approximately 2.0 m × 2.7 m per image (based on 3072 × 4096 resolution), resulting in an estimated spatial resolution of ~0.65 mm per pixel.

This allows the potential estimation of crack length, width, and area, adding engineering value to the dataset. While public datasets lack real-world spatial calibration, CrackVariety supports both qualitative detection and future quantitative analysis.

**Figure 1:**
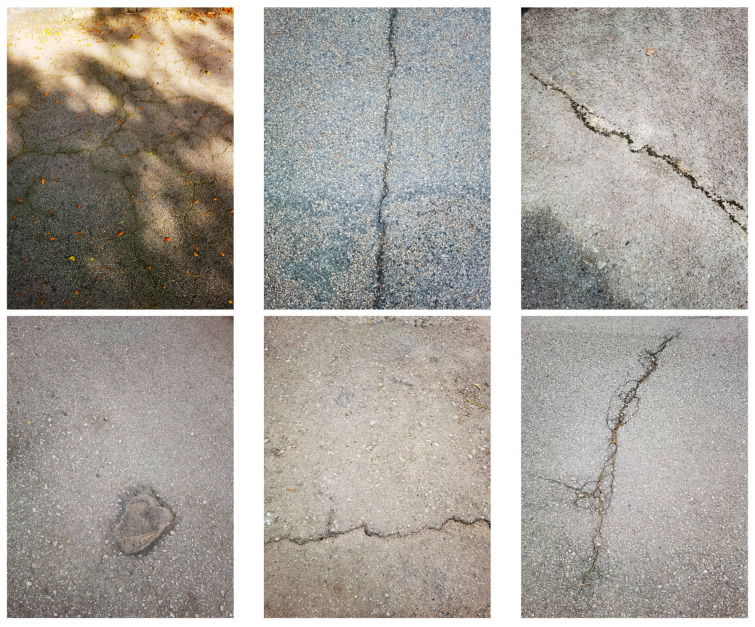
Sample images from the CrackVariety dataset.

Figure 1 presents representative sample images from the CrackVariety dataset. This dataset is publicly accessible for research purposes and can be obtained from https://github.com/Lucien430/CrackVariety-Dataset.git (accessed on 28 February 2025).

### 2.2. Data Preprocessing

To ensure dataset consistency, this study re-annotated crack types in publicly available datasets (CFD, CrackTree200, and Crack500), as well as the self-built dataset (CrackVariety), by using the LabelImg tool. All cracks were categorized into five types: LCs, TCs, ACs, OCs, and PHs. Image preprocessing plays a crucial role in improving crack detection accuracy by enhancing feature extraction. This study applied grayscale conversion and binarization to evaluate their impact on model performance. Grayscale conversion reduces computational complexity by eliminating redundant color information that may not contribute to crack detection. Previous studies [7] have demonstrated that grayscale processing enhances crack feature extraction when thresholding techniques are used. Binarization further enhances contrast between cracks and the background by converting images into a black-and-white format. Prior research [8] has shown that binarization improves model sensitivity to crack edges, particularly in high-contrast environments. These preprocessing techniques allow for a systematic comparison of their effects on detection performance, helping to determine the optimal input format for YOLOv8s-based crack detection.

#### 2.2.1. Grayscale Conversion

Various grayscale conversion methods were applied, each with distinct characteristics. The following sections describe these methods and their respective formulas.

(1) Average Method

One of the simplest grayscale conversion methods, the average method, computes the mean of the red (*R*), green (*G*), and blue (*B*) channels. This method is computationally efficient but preserves brightness and contrast poorly.

(1) $$Gray=\frac{R+G+B}{3}$$

(2) Green Channel Method

The green channel method extracts only the green channel for grayscale conversion, as human vision is most sensitive to green. This method retains more brightness details while maintaining computational efficiency.

(2) Gray=G

(3) Maximum Value Method

The maximum value method selects the brightest channel among *R*, *G*, and *B*. This technique enhances bright details, making it useful for applications requiring emphasized highlights.

(3) $$Gray=\max\left(R,G,B\right)$$

(4) Minimum Value Method

Conversely, the minimum value method selects the darkest channel. This method is beneficial when emphasizing dark regions within an image.

(4) $$Gray=\min\left(R,G,B\right)$$

(5) Weighted Average Method

The weighted average method adjusts RGB contributions based on human perceptual sensitivity, with green having the highest weight and blue the lowest. This method provides a balanced brightness representation and is widely used in computer vision.

(5) Gray=0.2989×R+0.5870×G+0.1140×B

**Figure 2:**
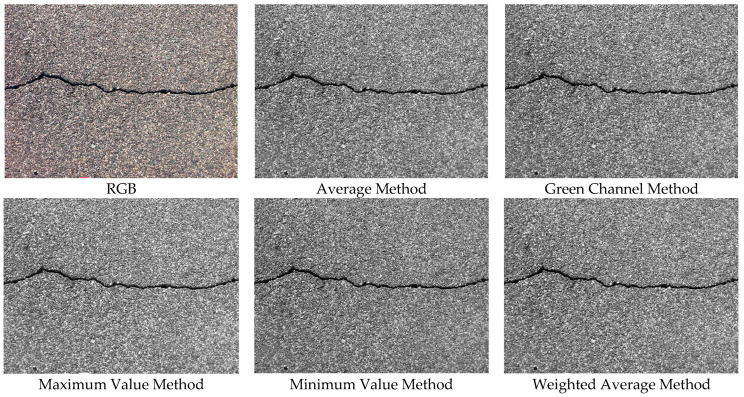
Different types of grayscale images.

Figure 2 shows the grayscale images generated using different methods. The detection performance of each grayscale format is compared for determining the optimal preprocessing approach.

#### 2.2.2. Binarization Conversion

**Figure 3:**
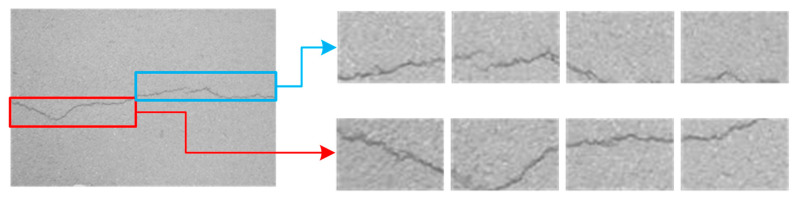
Cropped images.

**Figure 4:**
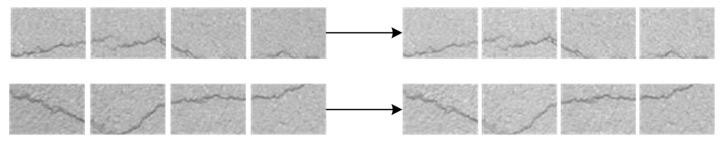
Compressed gray values.

**Figure 5:**
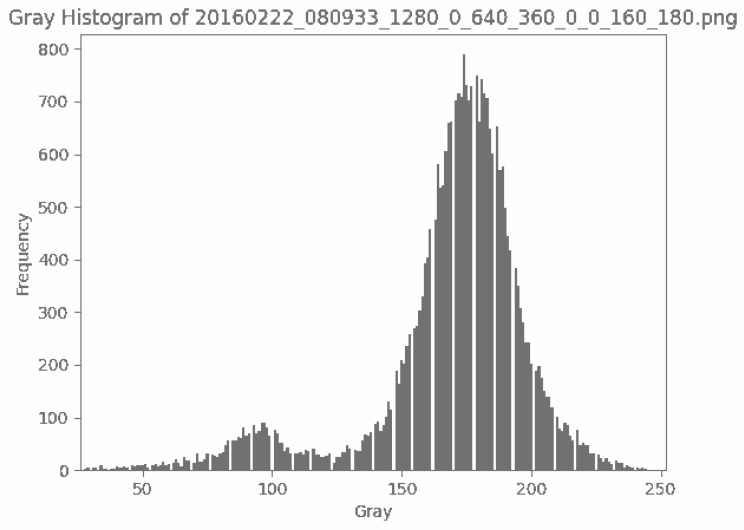
Gray histogram.

**Figure 6:**
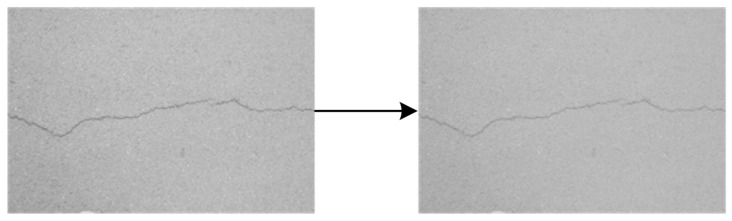
Grayscale compression of original image.

**Figure 7:**
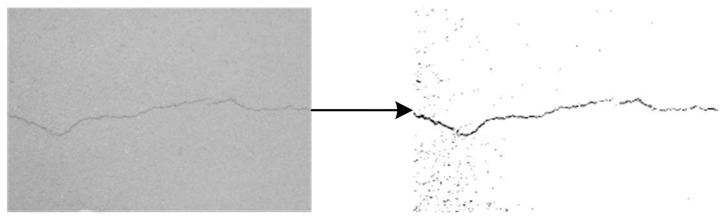
Binary image generation.

Binarization enhances crack background contrast. Figure 3, Figure 4, Figure 5, Figure 6 and Figure 7 illustrate the key steps of the binarization process, which are image cropping (Figure 3), grayscale range computation (Figure 4), histogram generation (Figure 5), grayscale adjustment (Figure 6), and final binarization thresholding (Figure 7). The process consists of seven steps:

1. Image cropping: to ensure a higher proportion of crack pixels, images are cropped to focus on crack regions (Figure 3).
2. Compute grayscale range: the brightness distribution of each cropped image is analyzed to determine its minimum and maximum grayscale values.
3. Grayscale compression: the grayscale range of the image is compressed to improve contrast for enhancing crack visibility (Figure 4).
4. Histogram generation: histograms are generated to visualize the grayscale value distribution for providing insights into contrast variations (Figure 5).
5. K-means clustering for threshold estimation:
  - Each image undergoes K-means clustering (k = 2), where pixels are grouped into two clusters representing cracks and the background.
  - The clustering is performed by minimizing the Euclidean distance between pixel values and cluster centers:
    (6) $$d\left(x_{i},c_{j}\right)=\sqrt{\sum_{k=1}^{n}{\left(x_{ik}-c_{jk}\right)}^{2}}$$
  - The binarization threshold for each image is determined as the midpoint between the two cluster centers.

6. Adjust grayscale in original image: the grayscale values in the original image are adjusted to match the contrast characteristics of the cropped regions (Figure 6).
7. Thresholding for binarization: the final threshold, computed as the average of all per-image thresholds, is applied to convert grayscale images into a binary format for ensuring a consistent processing standard across the dataset (Figure 7).

The implementation code of each step is provided in the Supplementary Materials for ensuring reproducibility and allows for further optimization of the binarization process.

### 2.3. Experimental Setup

Table 1 and Table 2 describe the hardware and software configurations used in the experiments.

### 2.4. Training Parameters and Evaluation Metrics

The YOLOv8s model was trained using the hyperparameters listed in Table 3. To ensure consistency, these settings were applied across all datasets. The batch size was set to 4 to ensure stable training under GPU memory limitations, while maintaining model convergence. The batch size was set to 4, while the number of epochs was 300. This choice was made to ensure sufficient model training while mitigating the risk of overfitting.

The performance of YOLOv8s-based crack detection was evaluated using standard object detection metrics, including Mean Average Precision (mAP), Precision, Recall, Inference Time, and FPS.

1. Mean Average Precision (*mAP*)

The primary metric used in this study was mAP@0.5, which calculates the mean of Average Precision (*AP*) across all crack categories at the IoU threshold of 0.5.

- *AP* for a single class is computed as

(7) $$AP=\sum_{n}\left(Recall_{n}-Recall_{n-1}\right)\times Precision_{n}$$

- *mAP* is then computed as the mean of *AP* values over all classes:

(8) $$mAP=\frac{1}{N}\sum_{i=1}^{N}{AP}_{i}$$

where *N* is the number of crack categories.

2. Precision and Recall

Precision and Recall measure model performance in terms of detection correctness.

- Precision (*P*): measures of quantity of detected cracks which are correct:

(9) $$Precision=\frac{TP}{TP+FP}$$

- Recall (*R*): measures of quantity of actual cracks which are successfully detected:

(10) $$Recall=\frac{TP}{TP+FN}$$

where *TP* (True Positive) refers to correctly detected cracks, *FP* (False Positive) refers to incorrectly detected non-crack regions, and *FN* (False Negative) refers to missed cracks.

3. Inference Time and Frames Per Second (*FPS*)

- Inference Time: measures the time taken to process a single image.
- *FPSs* is defined as

(11) $$FPS=\frac{1000}{InferenceTime}$$

*FPS* indicates the real-time capability of the model, with higher *FPS* values signifying faster processing speeds.

## 3. Results and Discussion

This section presents the experimental results obtained from YOLOv8s-based crack detection across four datasets: CFD, Crack500, CrackTree200, and CrackVariety. Each dataset was tested with three image formats (RGB, Grayscale, and Binarized). The grayscale images were generated using five different methods (average method, green channel method, maximum value method, minimum value method, and weighted average method). During the experiment, each dataset was divided into the training set and the validation set according to the ratio of 7:3. To ensure that the model generalizes well across different crack types, we employed a random stratified split, ensuring that each crack category (LC, TC, AC, OC, PH) was proportionally represented in both the training and testing sets. And we conducted multiple runs of the same experiment and reported the average performance across trials to mitigate potential bias. The evaluation metrics included mAP@0.5, Precision, Recall, Inference Time, and FPS.

### 3.1. Results and Discussion of CFD Dataset

**Figure 8:**
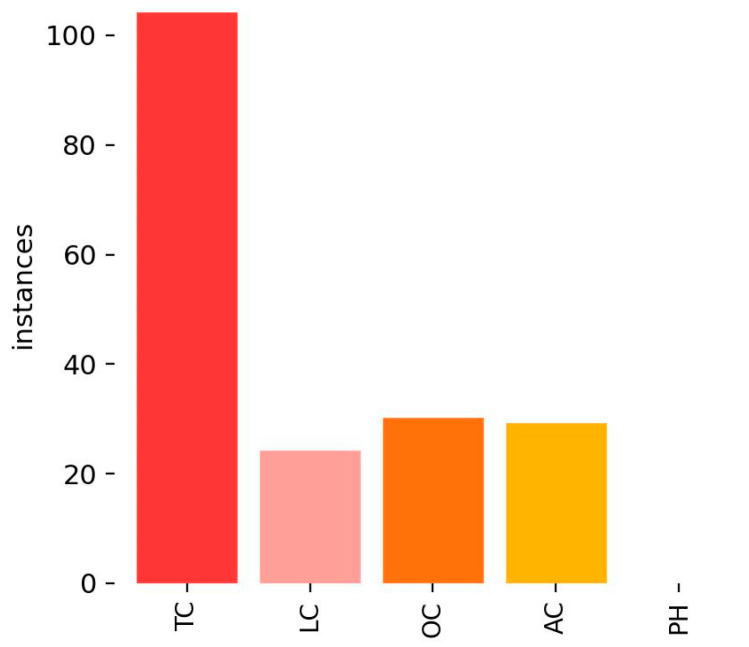
Label distribution of CFD.

This section presents experimental results and analysis for the CFD dataset. Figure 8 illustrates the distribution of crack labels in the CFD dataset. It is evident that TCs significantly outnumber other crack types, leading to a strong class imbalance. This imbalance may bias the model towards better detection of TCs while reducing its ability to accurately identify other crack types (e.g., LCs and ACs).

Table 4 summarizes the detection performance of YOLOv8s across different image types on the CFD dataset.

RGB images achieved the highest mAP@0.5 value of 0.222, confirming that color-based texture information is essential for YOLOv8s’s feature extraction. The minimum value method in grayscale achieved an mAP@0.5 value of 0.269, outperforming RGB. This suggests that enhancing darker regions may improve crack visibility. Other grayscale methods showed variable performance, with the weighted average method performing worse than expected (mAP@0.5 value of 0.229).

The minimum value method enhanced darker regions and made cracks more distinguishable. The green channel method performed moderately well and benefited from high brightness sensitivity in human perception but not necessarily in deep learning models. The weighted average method did not improve detection. This is an indication that human perceptual weighting may not align with YOLOv8s’s feature extraction mechanism.

The result of binarization image performance degraded (mAP@0.5 dropped from 0.222 to 0.165, which was the lowest value among all tested formats). The loss of grayscale texture and shading information negatively impacted detection, making it harder for the model to differentiate cracks from background noise.

**Figure 9:**
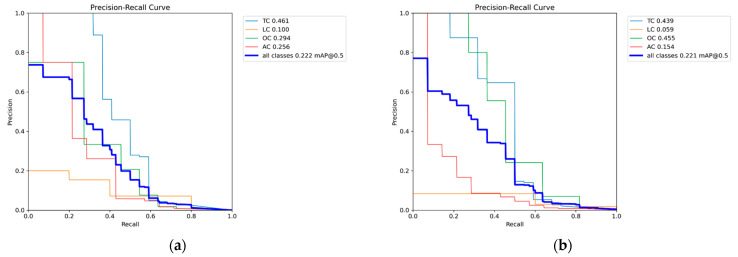

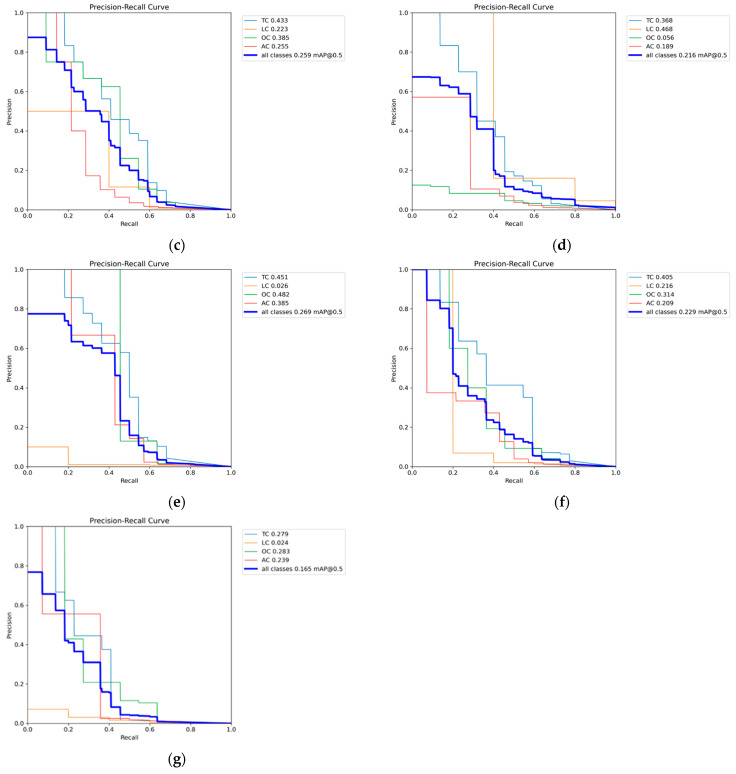
Precision–Recall curve of CFD dataset. (**a**) Precision–Recall curve for RGB images, (**b**) Precision–Recall curves for Average Method grayscale images, (**c**) Precision–Recall curves for Green Channel Method grayscale images, (**d**) Precision–Recall curves for Maximum Value Method grayscale images, (**e**) Precision–Recall curves for Minimum Value Method grayscale images, (**f**) Precision–Recall curves for Weighted Average Method grayscale images, (**g**) Precision–Recall curve for binarized images.

Figure 9a (Precision–Recall curve for RGB images) reveals that the TC category exhibited the highest detection accuracy, benefiting from its large representation in the dataset. The LC and AC categories demonstrated significantly lower recall, indicating that the model struggles with minority crack classes.

Figure 9b–f (Precision–Recall curves for grayscale methods) showed a similar trend, where TCs maintained higher detection rates, while LCs and ACs remained underrepresented.

Figure 9g (Precision–Recall curve for binarized images) demonstrated that detection performance degraded further, as the model failed to correctly differentiate minority classes from the background.

**Figure 10:**
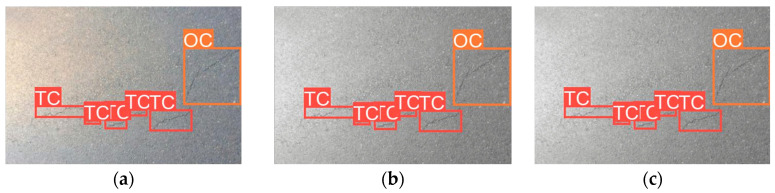

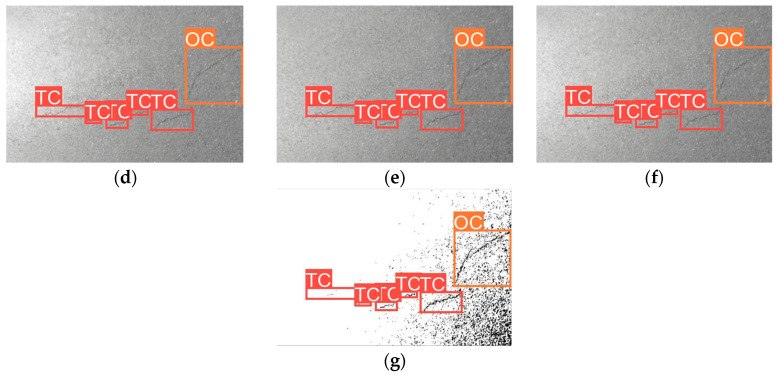
Detection results from the CFD dataset. (**a**) RGB images, (**b**) Average Method grayscale images, (**c**) Green Channel Method grayscale images, (**d**) Maximum Value Method grayscale images, (**e**) Minimum Value Method grayscale images, (**f**) Weighted Average Method grayscale images, (**g**) binarized images.

This confirms that dataset imbalance significantly impacts detection performance, as excessive bias towards one category limits YOLOv8s’s ability to generalize across different crack types. A more balanced dataset distribution is crucial for improving recall on underrepresented crack types. Figure 10 presents sample detection results from the CFD dataset. Subfigure (a) shows the detection result using RGB images, (b–f) illustrate the results from different types of grayscale images, and (g) displays the result when using binarized images.

### 3.2. Results and Discussion of Crack500 Dataset

**Figure 11:**
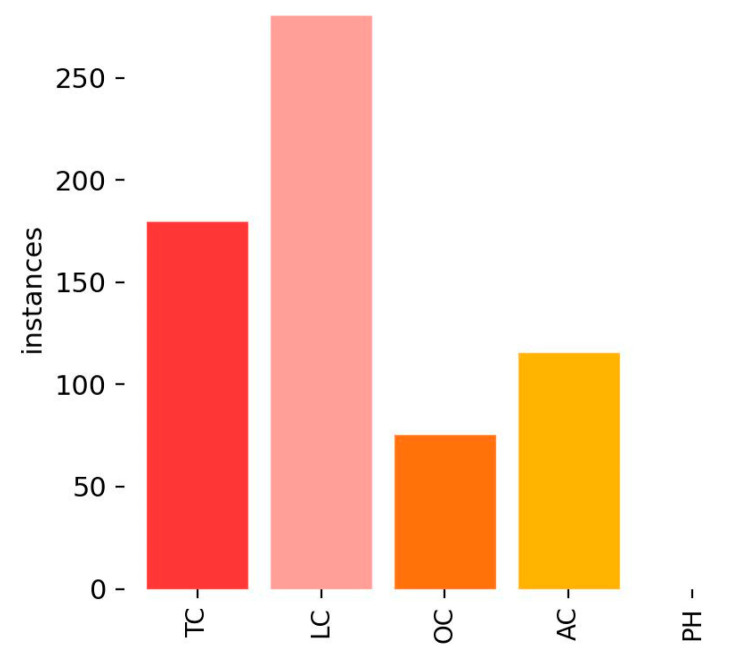
Label distribution of Crack500.

Figure 11 illustrates the distribution of crack labels in the Crack500 dataset. The dataset exhibited a noticeable class imbalance, where LCs were the most dominant category, followed by TCs. The significant disparity in crack type distribution may influence the model’s ability to generalize across all categories.

Table 5 presents the detection performance of YOLOv8s across different image formats for the Crack500 dataset.

RGB images provided the most stable performance, achieving an mAP@0.5 value of 0.384. The maximum value method achieved the highest grayscale mAP@0.5 value of 0.393, which is likely due to its ability to enhance high-contrast crack edges. The weighted average and average methods exhibited lower performances, which is possibly due to excessive smoothing that reduces fine structural details.

Binarization did not significantly degrade performance, which is likely due to Crack500’s highly uniform pavement texture. The mAP@0.5 for binarized images was 0.376. It is shown that while color information is beneficial, Crack500’s contrast stability enables relatively good performance even after binarization.

**Figure 12:**
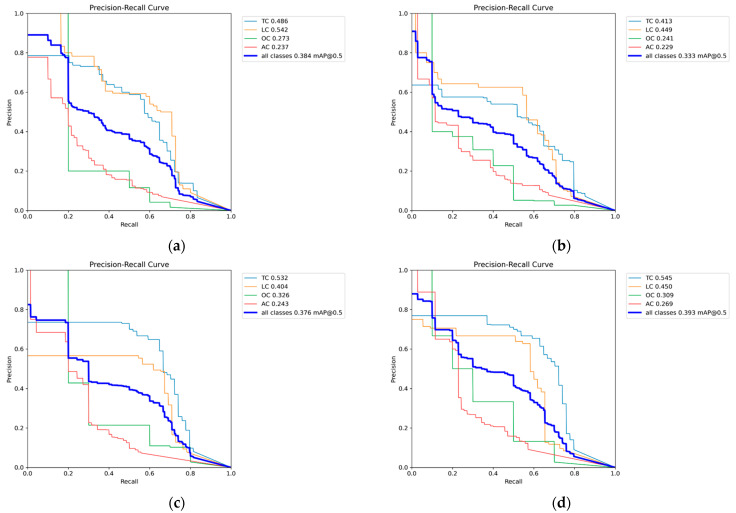

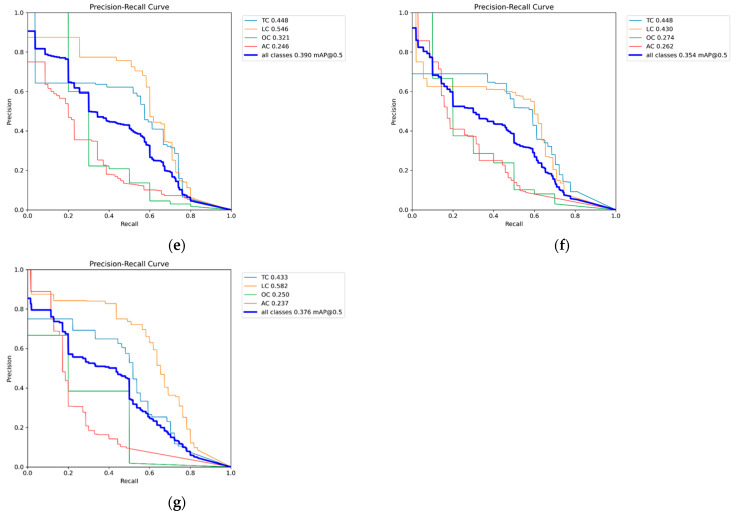
Precision–Recall curve of Crack500 dataset. (**a**) Precision–Recall curve for RGB images, (**b**) Precision–Recall curves for Average Method grayscale images, (**c**) Precision–Recall curves for Green Channel Method grayscale images, (**d**) Precision–Recall curves for Maximum Value Method grayscale images, (**e**) Precision–Recall curves for Minimum Value Method grayscale images, (**f**) Precision–Recall curves for Weighted Average Method grayscale images, (**g**) Precision–Recall curve for binarized images.

Figure 12a (Precision–Recall curve for RGB images) reveals that the TC and LC categories exhibited higher recall, which is likely due to their dominant representation in the dataset.

Figure 12b–f (Precision–Recall curves for grayscale methods) shows a similar trend, where TCs and LCs maintained higher detection rates, whereas underrepresented crack types (e.g., ACs) demonstrated significantly lower recall.

Figure 12g (Precision–Recall curve for binarized images) indicates that recall degradation was more severe in minority crack types, further reinforcing the negative impact of dataset imbalance.

**Figure 13:**
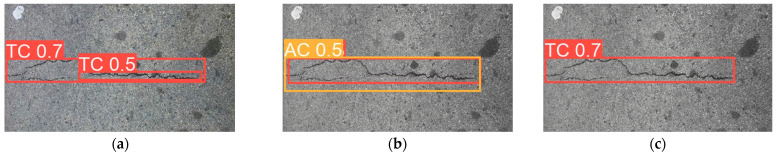

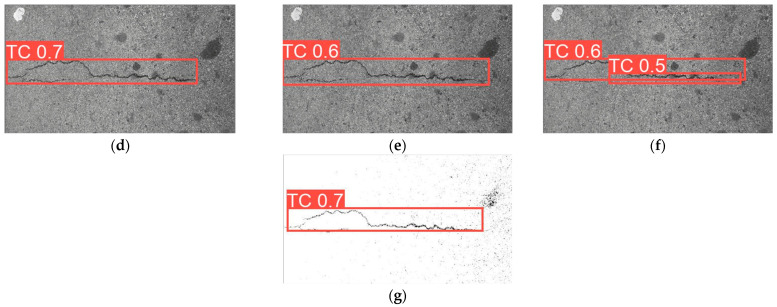
Detection results from the Crack500 dataset. (**a**) RGB images, (**b**) Average Method gray-scale images, (**c**) Green Channel Method grayscale images, (**d**) Maximum Value Method grayscale images, (**e**) Minimum Value Method grayscale images, (**f**) Weighted Average Method grayscale images, (**g**) binarized images.

These findings highlight that dataset imbalance significantly affects model performance, which led to higher recall for majority classes while minority classes suffered from lower detection accuracy. This suggests that dataset balancing strategies such as data augmentation or reweighting loss functions could be beneficial for improving model generalization across all crack types. Figure 13 presents sample detection results from the Crack500 dataset. Subfigure (a) shows the detection result using RGB images, (b–f) illustrate the results from different types of grayscale images, and (g) displays the result when using binarized images.

### 3.3. Results and Discussion of CrackTree200 Dataset

**Figure 14:**
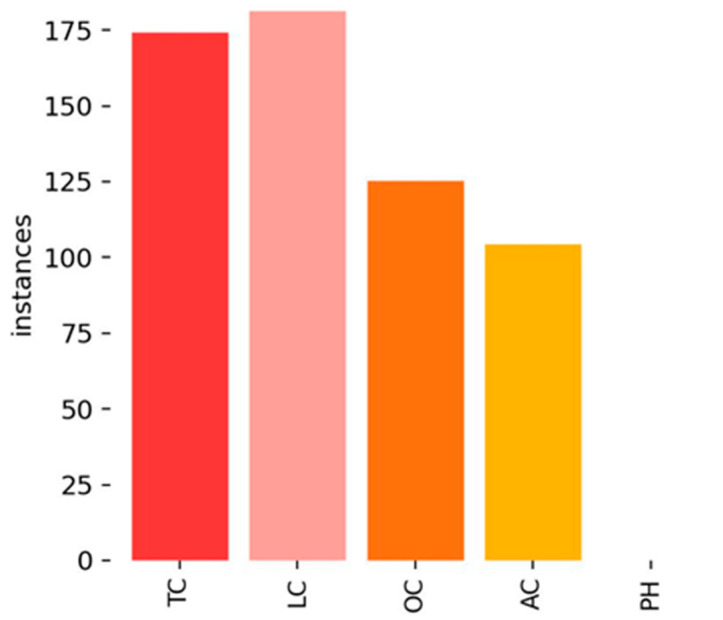
Label distribution of CrackTree200.

Figure 14 illustrates the distribution of crack labels in the CrackTree200 dataset. Similarly to CFD and Crack500, TCs and LCs (longitudinal cracks) dominated the dataset, which led to class imbalance. This imbalance may cause the model to favor these categories while reducing recall for underrepresented crack types such as ACs.

Table 6 presents the detection performance of YOLOv8s across different image formats.

RGB images provided a stable performance (mAP@0.5 value of 0.421), benefiting from color contrast information. The weighted average method achieved the highest grayscale mAP@0.5 value of 0.446, outperforming RGB. The green channel and minimum value methods showed slightly lower accuracy (mAP@0.5 value of 0.434), suggesting that balancing brightness contrast does not always enhance detection.

Binarization significantly reduced detection accuracy (mAP@0.5 value of 0.380). CrackTree200 contained more structured pavement textures, where grayscale variations are crucial for feature extraction. The binarization process eliminates these variations, making it harder to distinguish cracks from the background.

**Figure 15:**
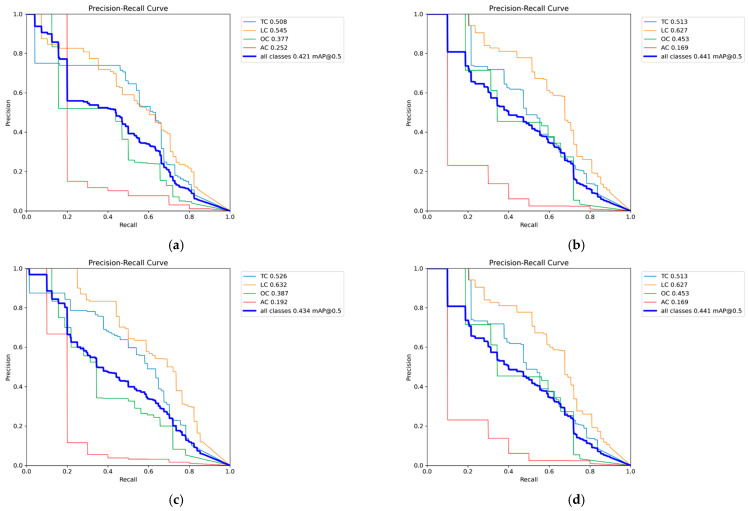

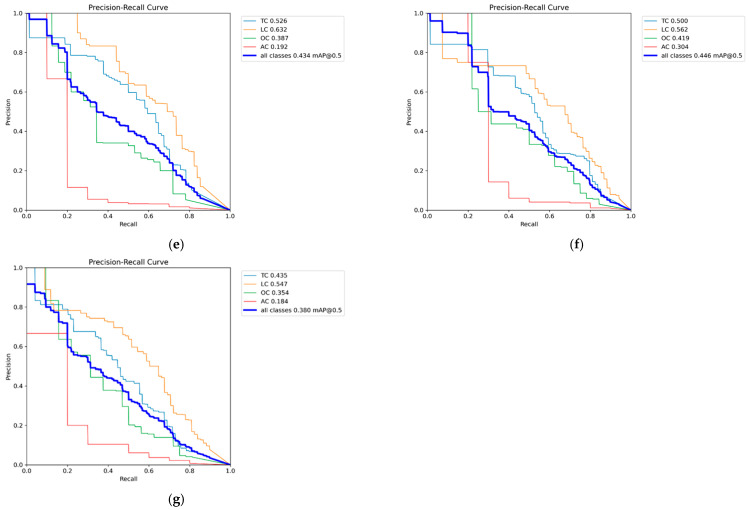
Precision–Recall curve of CrackTree200 dataset. (**a**) Precision–Recall curve for RGB images, (**b**) Precision–Recall curves for Average Method grayscale images, (**c**) Precision–Recall curves for Green Channel Method grayscale images, (**d**) Precision–Recall curves for Maximum Value Method grayscale images, (**e**) Precision–Recall curves for Minimum Value Method grayscale images, (**f**) Precision–Recall curves for Weighted Average Method grayscale images, (**g**) Precision–Recall curve for binarized images.

Figure 15a (Precision–Recall curve for RGB images) reveals that the TC and LC categories exhibited higher detection accuracy, which is likely due to their dominant representation in the dataset.

Figure 15b–f (Precision–Recall curves for grayscale methods) confirm a similar trend, where TCs and LCs maintained higher recall values, whereas AC showed significantly lower recall.

Figure 15g (Precision–Recall curve for binarized images) further demonstrates recall degradation, particularly for minority classes, suggesting that dataset imbalance negatively impacts model generalization.

**Figure 16:**
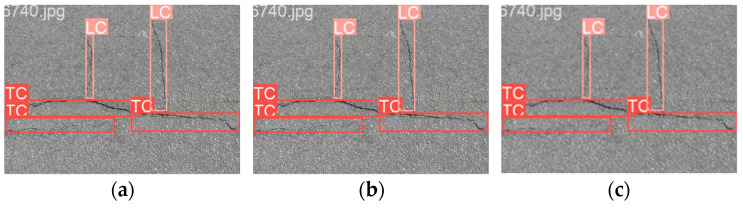

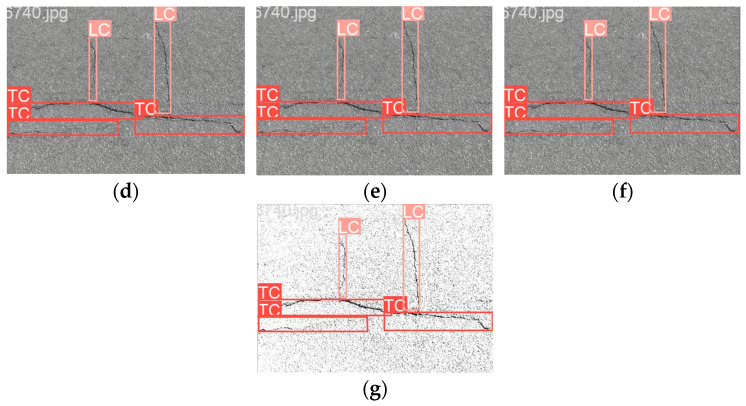
Detection results from the CrackTree200 dataset. (**a**) RGB images, (**b**) Average Method grayscale images, (**c**) Green Channel Method grayscale images, (**d**) Maximum Value Method grayscale images, (**e**) Minimum Value Method grayscale images, (**f**) Weighted Average Method grayscale images, (**g**) binarized images.

These findings emphasize that a more balanced dataset could improve detection performance for underrepresented crack types. The overrepresentation of TCs and LCs may cause the model to bias towards these categories, which led to reduced recall for minority classes like ACs. Figure 16 presents sample detection results from the CrackTree200 dataset. Subfigure (a) shows the detection result using RGB images, (b–f) illustrate the results from different types of grayscale images, and (g) displays the result when using binarized images.

### 3.4. Results and Discussion of CrackVariety Dataset

**Figure 17:**
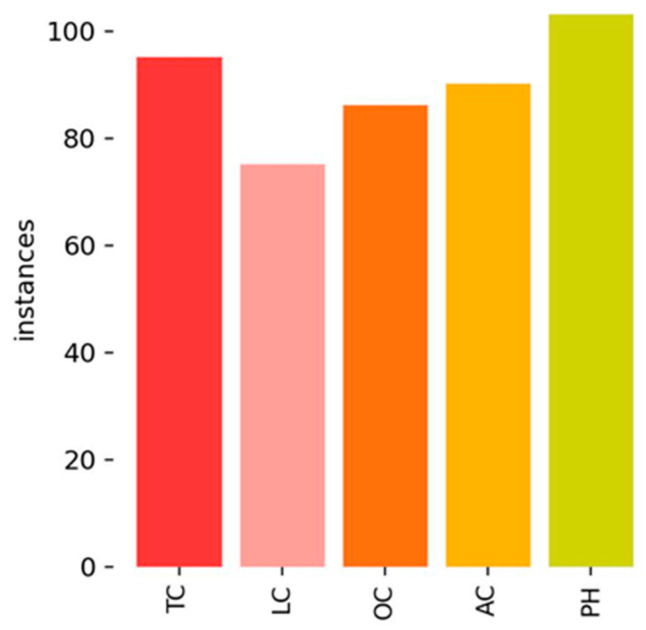
Label distribution of CrackVariety.

Figure 17 illustrates the distribution of crack labels in the CrackVariety dataset. Unlike the other datasets (CFD, Crack500, and CrackTree200), CrackVariety maintained a balanced distribution across all crack types that reduced the risk of class bias, allowing for a more generalized model evaluation.

Table 7 presents the detection performance of YOLOv8s across different image formats for the CrackVariety dataset.

**Figure 18:**
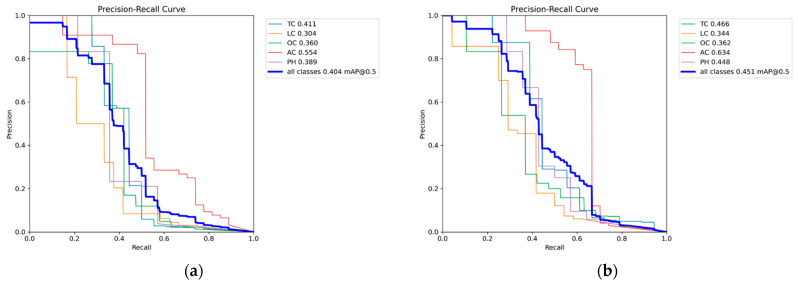

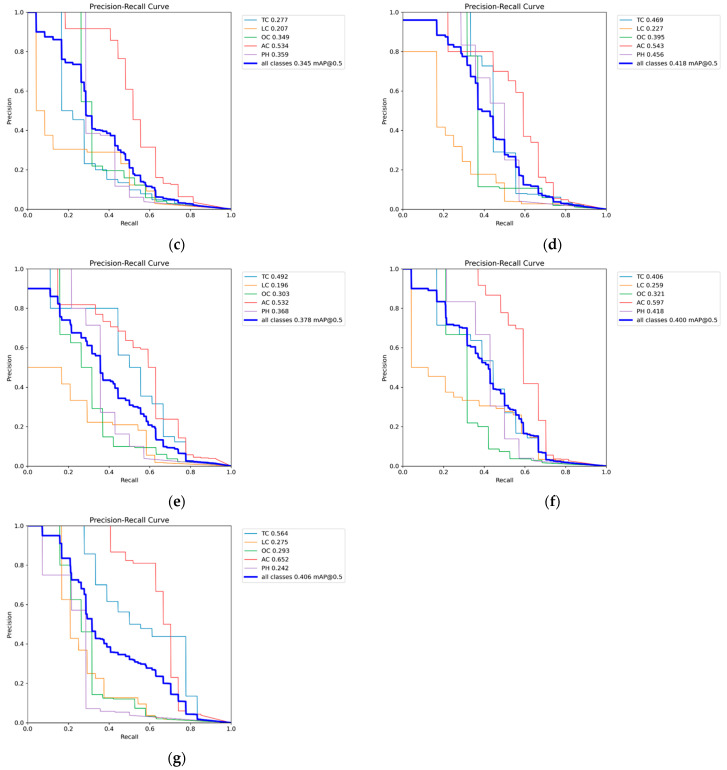
Precision–Recall curve of CrackVariety dataset. (**a**) Precision–Recall curve for RGB images, (**b**) Precision–Recall curves for Average Method grayscale images, (**c**) Precision–Recall curves for Green Channel Method grayscale images, (**d**) Precision–Recall curves for Maximum Value Method grayscale images, (**e**) Precision–Recall curves for Minimum Value Method grayscale images, (**f**) Precision–Recall curves for Weighted Average Method grayscale images, (**g**) Precision–Recall curve for binarized images.

Regarding the RGB image performance of CrackVariety, an mAP@0.5 value of 0.404 was achieved, which is higher than that of CFD (0.222) and Crack500 (0.384), but lower than that of CrackTree200 (0.421). The Precision–Recall curve (Figure 18a) reveals that ACs achieved the highest classification accuracy, while LC detection remained relatively weaker. Unlike other datasets, detection accuracy across different crack types was more balanced, benefiting from the dataset’s equal class distribution.

The average method achieved the highest grayscale mAP@0.5 value of 0.451, outperforming the RGB baseline. Other grayscale methods exhibited varying performances: the maximum value method (mAP@0.5 value of 0.418) and weighted average method (mAP@0.5 value of 0.400) performed moderately well. The green channel method resulted in the lowest grayscale detection performance (mAP@0.5 value of 0.345). These results suggest that brightness normalization (as performed in the average method) may enhance YOLOv8s’s feature extraction capabilities for CrackVariety.

The binarized image performance achieved an mAP@0.5 value of 0.406, which is slightly higher than that of RGB and comparable to that of the weighted average grayscale method. Unlike in other datasets, binarization did not lead to a significant accuracy drop, suggesting that CrackVariety’s balanced crack distribution helped stabilize model performance. The Precision–Recall curve (Figure 18g) confirms that binarized images performed better than expected, which is likely due to the equal representation of crack types that reduces class-dependent errors.

Figure 18a (Precision–Recall curve for RGB images) shows that detection accuracy across different crack types was more evenly distributed, which reinforces the advantages of dataset balance.

Figure 18b–f (Precision–Recall curves for grayscale methods) reveal that different grayscale conversions impacted feature extraction differently, with the average method performing the best.

Figure 18g (Precision–Recall curve for binarized images) demonstrates that binarization remained competitive with the grayscale and RGB formats, further supporting the importance of dataset structure in model performance.

**Figure 19:**
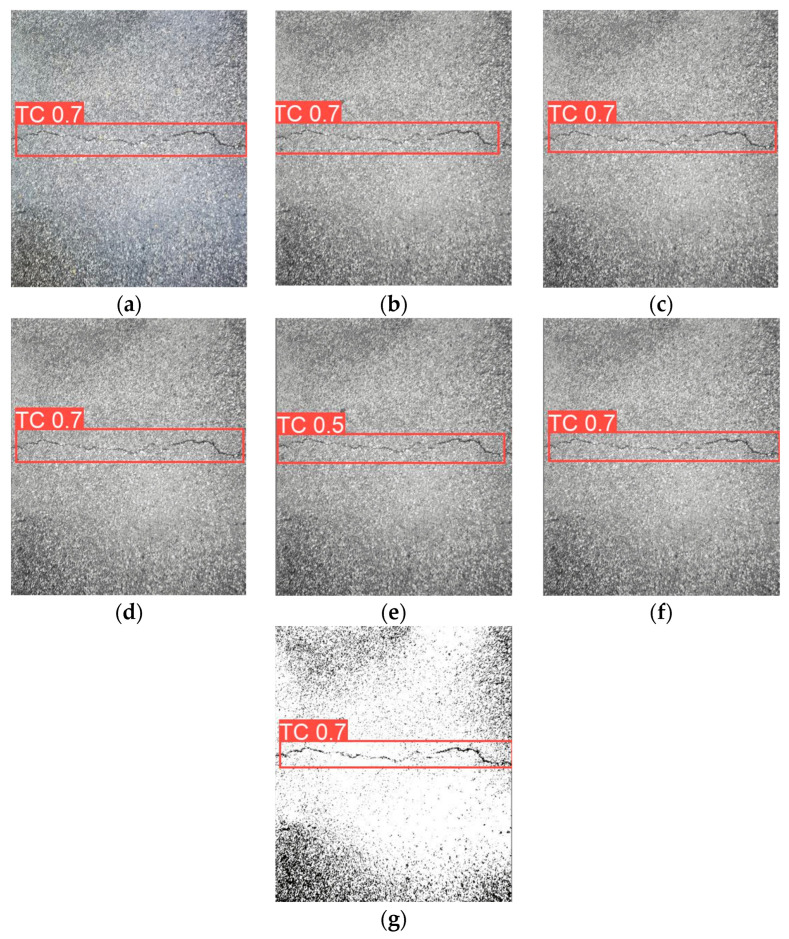
Detection results from the CrackVariety dataset. (**a**) RGB images, (**b**) Average Method gray-scale images, (**c**) Green Channel Method grayscale images, (**d**) Maximum Value Method grayscale images, (**e**) Minimum Value Method grayscale images, (**f**) Weighted Average Method grayscale images, (**g**) binarized images.

Figure 19 presents sample detection results from the CrackVariety dataset. Subfigure (a) shows the detection result using RGB images, (b–f) illustrate the results from different types of grayscale images, and (g) displays the result when using binarized images.

## 4. Conclusions

This study investigated the impact of image preprocessing methods and dataset balance on YOLOv8s-based road crack detection. Four datasets (CFD, Crack500, CrackTree200, and CrackVariety) were tested with RGB, grayscale (five conversion methods), and binarized images to evaluate their effects on detection performance.

1. RGB images consistently outperformed grayscale and binarized formats, confirming that preserving color-based texture and contrast enhances YOLOv8s’s detection accuracy.
2. Grayscale processing has different performance effects for different datasets.
3. Binarization generally degraded performance, except in CrackVariety (mAP@0.5 value of 0.406), where balanced crack distribution mitigated the negative effects of contrast loss.
4. Dataset size significantly affects model performance, as demonstrated by the CFD dataset, which exhibited the lowest mAP@0.5 value of 0.222 due to its limited number of images (118). This suggests that small datasets may lead to poor generalization and overfitting, emphasizing the need for a sufficient number of samples in training.
5. The reported FPS values across all experiments significantly exceed 30 FPS, ensuring real-time detection capability.
6. In CrackVariety, binarized images achieved relatively high detection performance (mAP@0.5 = 0.406) compared to other datasets. This may be attributed to the dataset’s balanced crack type distribution, which helps the model generalize better despite the reduced feature richness in binarized images.

For optimal detection accuracy, RGB images should be used in YOLOv8s-based crack detection models. Grayscale preprocessing should be dataset-specific, as no single method consistently outperformed RGB. Dataset balance is crucial for improving generalization.

This study has several limitations. First, the dataset scale, particularly for CFD (118 images), may affect the stability of conclusions regarding preprocessing effects. Second, although CrackVariety includes environmental diversity, this study did not explicitly analyze detection performance under each condition. Third, the detection results focus on crack classification rather than severity quantification or spatial dimension estimation, which are essential for practical road maintenance applications.

Future research can build upon this study in several directions. First, techniques such as data augmentation, resampling strategies, and loss function reweighting could be explored to mitigate the effects of class imbalance and improve model generalization. Second, the integration of multi-modal inputs (for example, combining RGB, grayscale, and edge-enhanced images) may further enhance detection robustness under varying pavement textures and visual conditions. Third, to improve real-world applicability, future studies should systematically evaluate model performance under diverse environmental conditions, including rain, shadows, oil stains, and occlusions. Fourth, both dataset structure and image preprocessing play important roles in crack detection performance. Future studies could investigate their relative contributions through controlled experiments to provide deeper insights into model optimization strategies. Fifth, deploying the detection framework in real-time road monitoring systems, potentially integrated with geographic information systems (GISs) and maintenance decision-making platforms, would significantly enhance its practical utility. Finally, incorporating crack length and width estimation as well as damage severity grading could transform the current detection pipeline into a comprehensive assessment tool for pavement health monitoring.

## Figures and Tables

**Table 1:** Hardware configurations used in the experiments.

Hardware	Configurations
CPU	Intel (R) Core (TM) i9-14900HX @ 2.20 GHz
GPU	NVIDIA GeForce RTX 4080 Laptop
RAM	32 GB

**Table 2:** Software configurations used in the experiments.

Software	Configurations
Operating System	Windows 11 Home Single Language
Development Environment	Anaconda 2.6.3, PyCharm Professional 2024.2.1
Programming Language	Python 3.9
Libraries and Frameworks	OpenCV 4.10.0 PyTorch 1.12.1 CUDA 11.6 cuDNN 8.9.7

**Table 3:** Hyperparameter settings used in this study.

Parameter	Value
Epochs	300
Input Image Size (imgsz)	640 pixels
Batch Size	4
Patience	300
Other Hyperparameters	Default YOLOv8 settings

**Table 4:** Experimental results of CFD dataset.

Datasets	Results	RGB	Grayscale					Binarized
			Average	Green Channel	Maximum Value	Minimum Value	Weighted Average	
CFD	Precision	0.387	0.230	0.403	0.365	0.435	0.297	0.197
	Recall	0.205	0.221	0.301	0.219	0.289	0.224	0.188
	mAP@0.5	0.222	0.221	0.259	0.216	0.269	0.229	0.165
	FPS	303.03	303.03	322.58	333.33	312.50	312.50	333.33

**Table 5:** Experimental results of Crack500 dataset.

Datasets	Results	RGB	Grayscale					Binarized
			Average	Green Channel	Maximum Value	Minimum Value	Weighted Average	
Crack500	Precision	0.386	0.504	0.516	0.543	0.591	0.491	0.483
	Recall	0.475	0.370	0.434	0.430	0.371	0.397	0.460
	mAP@0.5	0.384	0.333	0.376	0.393	0.390	0.354	0.376
	FPS	357.14	344.82	333.33	359.14	333.33	333.33	384.62

**Table 6:** Experimental results of CrackTree200 dataset.

Datasets	Results	RGB	Grayscale					Binarized
			Average	Green Channel	Maximum Value	Minimum Value	Weighted Average	
CrackTree20	Precision	0.593	0.455	0.608	0.455	0.608	0.612	0.504
	Recall	0.385	0.479	0.355	0.479	0.355	0.472	0.351
	mAP@0.5	0.421	0.441	0.434	0.441	0.434	0.446	0.380
	FPS	384.62	370.37	333.33	333.33	370.37	370.37	384.62

**Table 7:** Experimental results of CrackVariety dataset.

Datasets	Results	RGB	Grayscale					Binarized
			Average	Green Channel	Maximum Value	Minimum Value	Weighted Average	
CrackVariety	Precision	0.638	0.721	0.484	0.522	0.505	0.550	0.569
	Recall	0.368	0.392	0.338	0.403	0.393	0.375	0.364
	mAP@0.5	0.404	0.451	0.345	0.418	0.378	0.400	0.406
	FPS	222.22	263.16	192.31	222.22	222.22	227.27	222.22

## Data Availability

The raw data supporting the conclusions of this article will be made available by the authors on request.

## Supplementary Materials

The CrackVariety dataset and the implementation code for binarization conversion are available to download at https://github.com/Lucien430/CrackVariety-Dataset, accessed on 28 February 2025.

## Abbreviations

The following abbreviations are used in this manuscript:

PMS	Pavement Management System
DL	deep learning
YOLO	You Only Look Once
CNN	Convolutional Neural Network
CFD	Crack Forest Dataset
LC	longitudinal crack
TC	transverse crack
AC	alligator crack
OC	oblique crack
PH	pothole
mAP	Mean Average Precision
FPS	Frames Per Second
GISs	geographic information systems
